# Born small, die young: Intrinsic, size-selective mortality in marine larval fish

**DOI:** 10.1038/srep17065

**Published:** 2015-11-24

**Authors:** S. Garrido, R. Ben-Hamadou, A.M.P. Santos, S. Ferreira, M.A. Teodósio, U. Cotano, X. Irigoien, M.A. Peck, E. Saiz, P. Ré

**Affiliations:** 1 Instituto Português do Mar e da Atmosfera, Av. Brasília s/n, 1449-006 Lisboa, Portugal; 2 Marine and Environmental Sciences Centre, Faculdade de Ciências, Universidade de Lisboa, Campo Grande, 1749-016 Lisboa, Portugal; 3 Department of Biological and Environmental Sciences, College of Arts and Sciences, Qatar University, PO Box 2713, Doha, Qatar; 4 Centro de Ciências do Mar do Algarve, Universidade do Algarve. Campus de Gambelas, 8005-139 Faro, Portugal; 5 Marine Research Unit – AZTI Foundation, Herrera Kaia, Portualdea z/g, 20110 Pasaia, Spain; 6 Red Sea Research Center, King Abdullah University for Science and Technology, 23955-6900 huwal, Saudi Arabia; 7 Institute for Hydrobiology and Fisheries Science, Hamburg University, Olbersweg 24, 22767 Hamburg, Germany; 8 Institut de Ciències del Mar - CSIC, Ps. Marítim de la Barceloneta 37–49, 08003 Barcelona, Spain

## Abstract

Mortality during the early stages is a major cause of the natural variations in the size and recruitment strength of marine fish populations. In this study, the relation between the size-at-hatch and early survival was assessed using laboratory experiments and on field-caught larvae of the European sardine (*Sardina pilchardus*). Larval size-at-hatch was not related to the egg size but was significantly, positively related to the diameter of the otolith-at-hatch. Otolith diameter-at-hatch was also significantly correlated with survival-at-age in fed and unfed larvae in the laboratory. For sardine larvae collected in the Bay of Biscay during the spring of 2008, otolith radius-at-hatch was also significantly related to viability. Larval mortality has frequently been related to adverse environmental conditions and intrinsic factors affecting feeding ability and vulnerability to predators. Our study offers evidence indicating that a significant portion of fish mortality occurs during the endogenous (yolk) and mixed (yolk /prey) feeding period in the absence of predators, revealing that marine fish with high fecundity, such as small pelagics, can spawn a relatively large amount of eggs resulting in small larvae with no chances to survive. Our findings help to better understand the mass mortalities occurring at early stages of marine fish.

It is commonly accepted that mortality occurring during the early stages of development of marine fish (eggs through to post-larvae) is a major cause of the natural variation in the abundance of marine fish populations^1^,^2^,^3^. Recent findings suggest that the great majority of the mortality occurring at these early phases is selective, and that an individual’s phenotype influences its probability to survive (review in 4). Moreover, selection greatly varies amongst cohorts, implying that selective mortality is an important source of variability in total mortality, and thus in the variability in recruitment strength^4^. Therefore, understanding the phenotypic variability in selected traits is fundamental to understanding the dynamics of fish populations and anticipating which species/populations may be most affected by environmental change.

The high mortality experienced during the larval phase of fishes is widely regarded to be size-dependent^5^. In the growth-dependent mortality hypothesis, faster-growing individuals within a population have lower risks of mortality due to predation since i) larger larvae have stronger swimming capabilities than smaller ones, and ii) fast-growing larvae experience a shorter duration of the larval period, decreasing the period of time when they are most vulnerable to predators. Although there is a fair amount of field and laboratory research showing that the mortality of fish larvae is growth-dependent (e.g.^6^,^7^,^8^), most previous studies have focused on how extrinsic factors such as adverse environmental conditions impact survival by reducing larval growth (size-at-age). However, other intrinsic factors such as inter-individual differences in metabolic rates and maternal energy provisioning may also impact on the size, growth and survival of larvae, even when individuals are faced with optimal environmental conditions. Thus intrinsic and extrinsic factors combine to affect growth and survival^10^.

Generally, larger size-at-hatch has been associated with higher survival probabilities in fish (e.g.^11^,^12^,^13^) especially under adverse environmental conditions^14^. Given that even small differences in mortality rates experienced during the larval period can drive large fluctuations in the recruitment of marine fishes^15^,^16^, variation in early life-history traits such as size-at-hatch can potentially explain a large portion of that variability^17^. Size-at-hatch depends on extrinsic and intrinsic factors such as temperature, additive genetic variance and maternal effects^17^ as well as their interaction^18^. Temperature influences yolk utilization efficiency which affects the size obtained by larvae at the end of endogenous feeding (e.g.^19^,^10^). After fertilization, not all embryos synchronously hatch and those hatching later are normally larger but also may have fewer yolk reserves than earlier-hatching smaller larvae^20^,^21^. In several batch-spawning marine fishes, maternal effects have been reported to influence larval size and viability, e.g. larger females spawn larger eggs than smaller females (e.g.^22^,^23^,^24^) and larger eggs lead to larger larvae or larvae with higher nutritional quality (e.g.^25^,^26^). The opposite has also been reported for the damselfish (*Pomacentrus amboinensis*), for which smaller females produced larvae of longer length and larger energy reserves at hatching^27^. Paternal (i.e. genetic) effects are also important in determining larval size, e.g. significantly influence larval length and yolk-sac volume in the Atlantic herring (*Clupea harengus*)^28^,^29^.

The European sardine *Sardina pilchardus* is one of the most abundant small pelagic fish species in the Northeast Atlantic and the Mediterranean^30^,^31^, dominating the ichthyoplankton community in the Western Iberian upwelling ecosystem, particularly during the colder months of the year^32^. In recent years, the Atlanto-Iberian sardine stock has displayed large fluctuations in size and spatial distribution and has now reached historically minimum values of population abundance and recruitment strength^33^. Population fluctuations of short-lived small pelagic fishes are known to result from recruitment variability^34^, and changes in prey availability are considered to be a main cause of such variability, either by affecting the intensity of reproduction or by directly impacting the growth and survival of early life stages^35^. Trophodynamically-mediated successful reproduction and/or larval survival promote strong year classes, preventing the collapse of small pelagic fish stocks, which often has dramatic socio-economic consequences^36^.

The main objective of this work was to assess the relation between size-at-hatch and early viability of the larvae of European sardine. Our analysis included both wild individuals collected from the field and sardine larvae reared in the laboratory under different feeding conditions. The second objective was to study whether egg size influenced the size-at-hatch of sardine larvae. Our main tool was the microstructure analysis of larval otoliths, a technique previously used to examine the growth-selective mortality hypothesis in marine fish larvae (e.g.^37^,^38^,^9^). Unveiling the relationship between size-at-hatch and survival potential in the laboratory and field will help clarify whether this intrinsic factor plays a direct role in the mortality experienced by sardine larvae and the potential for year-class success.

## Results

### Egg size and sardine larvae size-at-hatching

**Figure 1:**
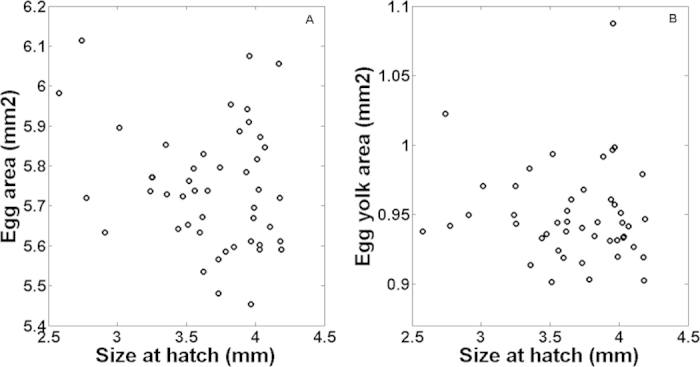
Relationship between sardine larvae size-at-hatching and egg size (panel A) and egg yolk-sac area (panel B).

The size of the sardine eggs incubated individually (Experiment 1), estimated from the area of the egg, ranged from ~5.4 to 6.1 mm^2^ (mean: 5.7 ± 0.17 SD mm^2^). The average yolk-sac area of the eggs was 0.9 ± 0.03 SD mm^2^. Larval total length-at-hatch varied from 2.57 to 4.18 mm, with a mean of 3.6 ± 0.37 SD mm (n = 47). No statistically significant relationship was found between either egg size or yolk-sac area and the total length-at-hatch of sardine larvae (simple linear regression analysis, *P*-values = 0.16 and 0.52, respectively; Fig. 1).

### Sardine larvae development and mortality

Approximately 72 h after spawning, sardine eggs had already hatched and the mean (±SD) larval length-at-hatch was 3.6 ± 0.34 mm (n = 73). Individual larvae ranged from 2.8 to 4.2 mm at hatch. Larvae hatched with no eye pigmentation and a closed mouth. Larvae started exogenous feeding at an age of 4 days post-hatch (dph).

**Figure 2:**
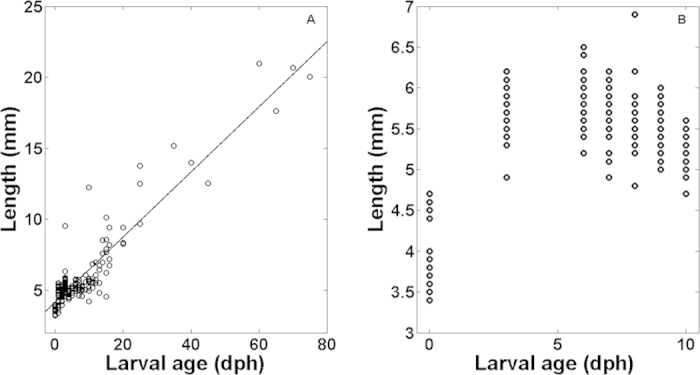
Growth of sardine larvae reared in the laboratory under starvation (panel A) and under optimal feeding conditions (panel B).

When reared under optimal feeding conditions (Experiment 2), larval size (total length) increased linearly throughout ontogeny (Fig. 2A; Supplementary Table S1 online) following the linear regression equation (n = 213):


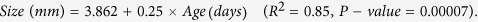


When reared with no prey (Experiment 3), sardine larvae were able to grow in length only during the endogenous feeding period (<5 dph) and then the mean size-at-age declined by 9.7% from 6 dph to 11 dph when all larvae died (Fig. 2B).

**Figure 3:**
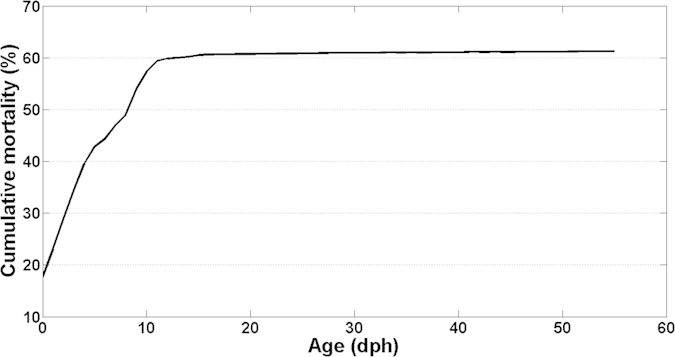
Cumulative mortality (%) through ontogeny of sardine larvae reared in laboratory conditions at 15 °C and salinity of 35 under defined optimal feeding conditions.

Total larval mortality during the experimental period for larvae reared with optimal feeding conditions was 61.3%, and mostly occurred during the first 10 dph (57.3%). In particular, larvae mortality concentrated during the first 5 days after hatch, comprising 42.8% of the total (cumulative) larval mortality experienced during the whole experimental period (Fig. 3). These first 5 dph correspond to an exclusively endogenous feeding period until 2 dph, when total mortality was 34.5% and a period of mixed endogenous and onset of the exogenous feeding period (3 to 5 dph), when mortality was 8.3% of the total cumulative mortality during the experimental period.

### Hatch check diameter and size-at-hatch for larvae reared in the laboratory under optimal feeding conditions

**Figure 4:**
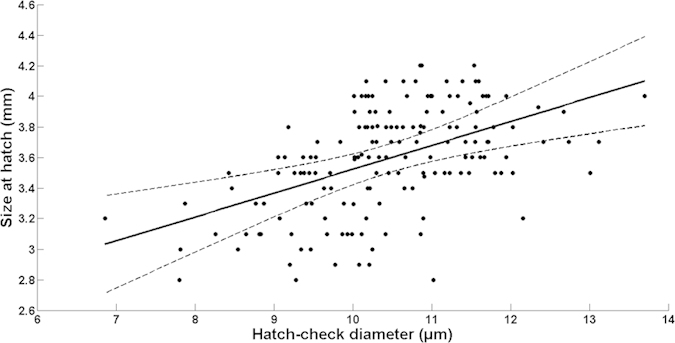
Relationship between larvae size-at-hatch (total length, mm) and otolith hatch check diameter (μm) for sardine larvae reared in the laboratory under optimal feeding conditions.

For each of the 820 sardine larvae reared under optimal feeding conditions (Experiment 2), we extracted one or (when possible) both sagittal otoliths for analysis. From the 184 individuals sampled at the day of hatching, a clear hatch check was present in 161 larvae, with a diameter of 10.4 ± 1.11 μm (mean ± SD). The relationship between larval size at hatch (*L*_*0*_) and otolith hatch check diameter (*HCD*) was best described by a simple linear regression model (Fig. 4; Supplementary Table S1 online):


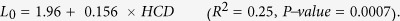


### Hatch check diameter and survival for larvae reared in the laboratory under optimal feeding conditions

The hatch check was clearly visible in the sagittal otolith of 608 individuals (70% of all sardine larvae reared under optimal feeding conditions). The hatch check diameter of the otoliths of larvae sampled throughout the laboratory rearing period was 11.4 ± 1.4 μm (mean ± SD).

**Figure 5:**
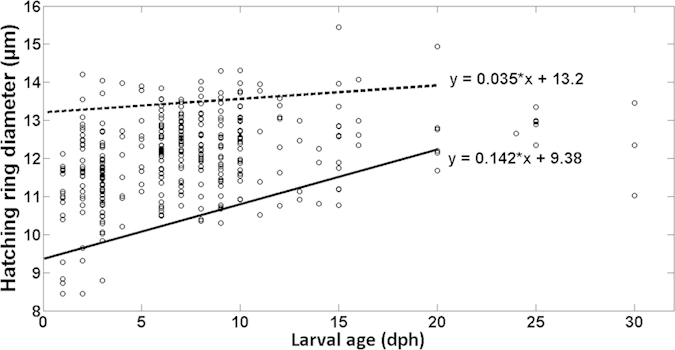
Relation between sardine larvae age (days post-hatch) and otolith first increment check diameter (μm) for European sardine (*Sardina pilchardus)* larvae reared in the laboratory under optimal feeding conditions.

Changes in the distribution of the hatch check diameters versus larval age (Fig. 5) indicated that larvae with smaller hatch check diameters disappeared from the population significantly faster than larvae having larger hatch check diameters. Quantile regression analysis confirmed that the slope of the regression using the lower, 10^th^ percentile of diameters was positive and statistically different from 0 (+0.142, *P-value* = 0.00003; Supplementary Table S1 online), indicating that larvae having relatively small hatch check diameters disappeared from the population through time. In contrast, the slope of the regression for the upper, 90th percentile was not significantly different from zero (+0.035, *P-value* = 0.013; Supplementary Table S1 online), indicating that larvae with larger hatch check diameter were not removed from the population through time.

### Hatching check diameter and viability for larvae reared under starvation

**Figure 6:**
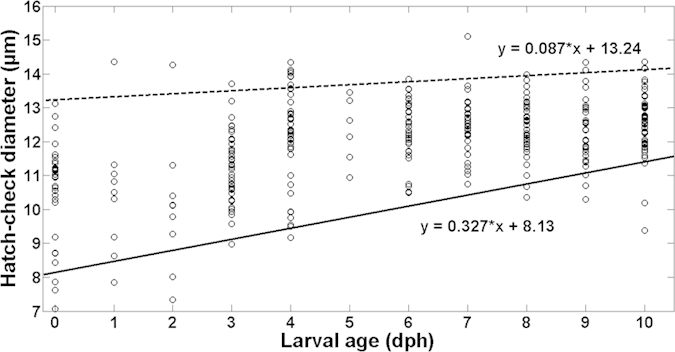
Relation between sardine larvae age (days post-hatching) and otolith first increment check diameter (μm) for European sardine (*Sardina pilchardus)* larvae reared in the laboratory under starvation.

Regarding the larvae reared in starving conditions, it was possible to extract the otoliths of 391 out of the 430 larvae sampled. From the otoliths corresponding to the larvae sampled at the day of hatching (35 individuals), 28 had a clear hatch check. Average hatch check diameter of larvae reared under starvation was 11.9 ± 1.33 μm (mean ± SD). Larvae with smaller hatch check diameters disappeared from the population significantly faster than larvae having larger hatch check diameters, particularly from age 3 onwards (Fig. 6). Quantile regression analysis confirmed that the slope of the lower, 10^th^ percentile fit was positive and statistically different from 0 (+0.327, *P-value *= 0.000002; Supplementary Table S1 online), indicating that larvae provided with small hatch check diameters were disappearing from the population through time. In contrast, the regression slope for the 90th percentile was not statistically different than zero (+0.087, *P-value* = 0.13; Supplementary Table S1 online), indicating that larvae with larger hatch check diameters remained within the population.

### Otolith hatch check diameter and age of wild sardine larvae from the Bay of Biscay

**Figure 7:**
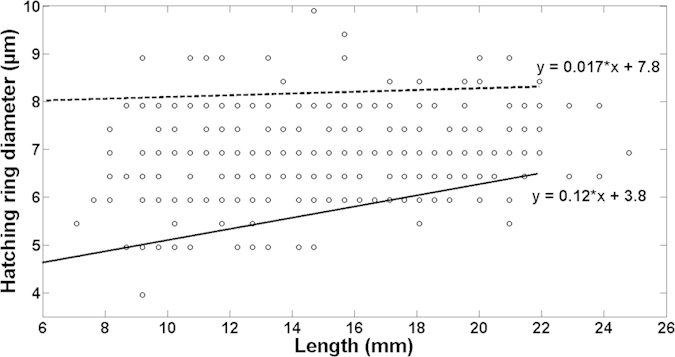
Relation between sardine larvae age (days post-hatching) and otolith first increment check diameter (μm) for larvae captured in the Bay of Biscay.

The otoliths of 492 sardine larvae collected in the Bay of Biscay were analysed. Sardine larvae ranged from 2.6 to 24.8 mm in length and had between 0 and 28 otolith increments. Hatch check radius was measured for all larvae, ranging from 3.9 to 9.9 μm. Similarly to what was observed in the reared sardine larvae, in the wild sardine larvae there appeared to be a positive linear relationship between hatch check size (radius) and age of the larvae (Fig. 7). Quantile regression analysis shows that this pattern result from the gradual loss of relatively small size-at-hatch larvae from the population (10^th^ quantile regression slope +0.12, *P-value* = 0.039; Supplementary Table S1 online) and a relatively stable contribution of larvae with larger hatch check diameters in the population (i.e, the upper, 90th quantile regression fit shows no statistically significant slope (+0.017, *P-value* = 0.045; Supplementary Table S1 online).

## Discussion

Our study provides solid evidence that the survival probability of fish larvae, both for larvae reared in the laboratory and for wild specimens, is closely linked to the size of the larvae at hatch. The first otolith check, formed at hatching, was linearly related to the larval size-at-hatch, and larvae surviving past the first-feeding stage had higher mean hatch-check diameters when compared to those analysed at the day of hatching. This fact means that larvae that are larger at hatch have higher chances to survive.

This size-dependent mortality cannot simply be driven by extrinsic factor such as predation, since our laboratory experiments did not include predators. One could argue that the size-selective mortality observed in the laboratory experiments when larvae were provided prey is a consequence of larger larvae being superior feeders and utilizing a broader prey size spectrum and, thus, tending to grow faster than smaller larvae. However, a considerable amount (34.5%) of the total larval mortality occurred before the age of 3 dph, which falls within the exclusively endogenous feeding period while an additional 8.3% occurred at 3 and 4 dph, a period of mixed endogenous and exogenous feeding (at 15 °C, sardine larvae start exogenously feeding at 3 to 4 dph). In comparison, only 15% of the total mortality occurred at first-feeding and only 2% occurred for larvae ≥10 dph. Correspondingly, the selective disappearance of relatively small larvae at hatch was noticed during the endogenous feeding period and early start of the exogenous feeding (<5 dph) and after that period no larvae with hatch-check diameters smaller than 10 μm were found. In the absence of prey, larvae did not live more than 12 dph and larvae with relatively small hatch size died earlier than larvae that were relatively large at hatch.

Size-at-hatch can be the result of maternal or intrinsic (metabolic) factors and otolith growth is directly related to the metabolic rate and is only indirectly correlated with somatic growth; therefore the hatch-check area (measured in this study) is likely a proxy for embryonic metabolic rate. Larger larvae at hatch correspond to individuals with a relatively high metabolic rate^39^ while larvae that are smaller at hatch have lower metabolic rate during the embryonic stage than larger larvae^40^. We speculate that size-selective mortality during the endogenous feeding period most likely stems from metabolic differences arising during early embryonic development, meaning that sardines with small size-at-hatch are unable to grow and survive due to metabolic limits and the high costs of development in young larvae. Another hypothesis to explain the pattern found in this study would be that larger larvae correspond to those with more yolk reserves, as shown e.g. for capelin, *Mallotus villosus*^41^, and the small larvae with higher mortalities are below a given threshold of reserves where embryonic or early life development fail. In fact, it is generally accepted that females producing eggs with higher yolk reserves would have progeny which were larger-at-hatch and with an increased chance of survival. In our laboratory experiments, we found that egg size and egg yolk content were not significantly related to the size-at-hatch of the sardine larvae. However, maternal effects have indeed been shown to influence egg quality in wild sardine populations^42^,^43^. Moreover, in wild populations, the viability of larval sardines has been shown to depend on the quality of the eggs produced, particularly their protein content^44^. On the other hand, size-selective mortality that occurred during the mixed feeding period might have been related to the lower feeding abilities of the smaller larvae. In fact, it has been demonstrated that size-selective mortality of first-feeding larvae is related to the inefficiency of small larvae to capture prey, even if present in high concentrations, due to limited feeding performance at low Reynolds numbers (“hydrodynamic starvation”^45^).

A large number of field studies has demonstrated that larger larvae at a given age have a survival advantage in terms of feeding success and predator avoidance (e.g.^46^,^47^) because they swim faster and have a broader prey size spectrum. Particularly for sardines, back-calculated otolith radius-at-age suggests the existence of growth-selective mortality^9^. On the other hand, it has been demonstrated for the radiated shanny (*Ulvaria subbifurcata*), that larvae with small otolith size-at-hatch and low metabolic rates survived longer than larger larvae that consumed metabolic fuel at higher rates^40^, but such mortality pattern was only apparent when food was limiting. According to the authors, the advantage of producing small larvae was related to those species facing variable feeding conditions, where relatively slow-growing, small larvae would consume their yolk reserves more slowly, extending the endogenous period and increasing their chances to encounter adequate food patches. Therefore, the higher the unpredictability of the environment, the higher the variability of hatch-check size (e.g. intrinsic metabolic rates). Sardines are mostly abundant in productive upwelling ecosystems, where plankton productivity is highly variable, both spatially and temporally. European (and other) sardine larvae are fast-growing and require high prey concentrations for survival^48^. Finding these prey patches by active foraging is enhanced starting at 20 dph, when larvae start to actively swim^49^. Furthermore, swimming ability was positively correlated to size-at-age^49^, which can explain why larger sardines are favoured in the wild. In fact, the high genetic correlation between larval size and swimming performance suggests that direct selection on swimming performance likely induces an appreciable, correlated response in larval size-at-hatch^17^.

A key question is: if larger and fast-growing larvae are always favoured in relation to smaller and slow-growing larvae, irrespective of feeding conditions, then why should the small progeny be produced? According to^50^, selection operates on overall fitness and other traits influencing overall life-time mortality (but are associated with smaller size-at-hatch) must also be considered. For example, it has been shown that mean offspring size evolves to a value that maximizes maternal fitness (i.e. selection of maternal fecundity) even though mean offspring survival might be much less than optimal^10^. To our knowledge, there are no studies demonstrating a trade-off between the number and size of offspring for sardines. However, according to^10^, evolutionary responses of larvae would likely be balanced by reproductive selection favouring mothers that produce more, smaller offspring. Therefore, female sardines may trade-off smaller larval size for higher fecundity, even if it is done at the expense of decreasing the probability of survival for a proportion of her offspring.

The existence of selective mortality can be an important source of bias on the estimation of growth^51^. For a tropical clupeid, the silver-stripe round herring *Spratelloides gracilis*^52^, selective mortality only acted on one out of six cohorts examined. In that study, selective mortality occurred from 0 to 15 dph in a cohort of particularly small and slow-growing larvae, which increased the mean size-at-age in older larvae to values above that of cohorts that had not undergone this process. It is important to note that the growth rate bias (overestimation) caused by size-selective mortality is likely greatest when cohorts are growing under sub-optimal environmental conditions (e.g. too cold or warm temperatures) which cause higher mortalities of small larvae. Therefore, in some cases it will be essential to parametrize size-selective mortality if one hopes to generate robust growth functions for fish larvae.

Fish experience massive mortalities during the first phases of development, which represents a key time for phenotypical selection to take place. Although not all mortality is selective, recent findings have shown that the majority of the mortality occurring at the early stages of marine fish larvae, either during the embryonic or during the onset of the exogenous feeding, is selective^4^. The distribution of phenotypes that affect early life survival vary substantially among cohorts, implying that the strength and direction of the selection also vary significantly^4^. This explains the wide range relationships reported between larval size-at-hatch and selective mortality, ranging from undetected size-selective mortality for a specific cohort (Atlantic herring, *Clupea harengus*^53^) to strong selection of larger larvae-at-hatch (Atlantic cod, *Gadus morhua*^13^,^14^). Size selective mortality seems to be particularly intense under adverse environment^14^, revealing the importance of food availability for early larvae, when small larvae with lower feeding abilities^45^ are forced to forage intensely to sustain high growth rates, at the cost of increased predation risk^54^ other factors can also influence larval size-at-hatch, and therefore influence the direction and intensity of selective mortality, and these include temperature^55^ and size-selective fishery pressure, affecting both maternal and larvae size^56^. Given the importance of larval survival to fish population dynamics, it is essential to understand the different causes of larval mortality and the combined influence of intrinsic and external factors in shaping selective mortality, which are responsible for the natural variations of recruitment strength for marine fish populations.

## Material and Methods

### Sardine rearing and experimentation

The experimental procedures used in this study were approved by the Animal Welfare and Protection of the General Direction of Veterinary (DGV) of the Ministry of Agriculture, Rural Development and Fisheries, approval number 0420/000/000/2011, Lisbon, Portugal, and were in accordance with the guidelines of the European Directive 2010/63/EU of September, 22 and the Portuguese legislation that implements such directive nationally.

Sardine larvae were hatched from eggs spawned by captive sardines (*Sardina pilchardus*), captured by purse-seiners in western Portugal (Peniche) during 2009 and 2010, and kept in a 15000-L cylindrical tank at the Lisbon Aquarium (*Oceanário de Lisboa*). Adult fish started spawning naturally by adjusting the photoperiod and temperature to natural conditions. The eggs for the experiments were collected from the captive brood stock by a 500-μm mesh bag placed in the skimmers of the tank.

We conducted 3 experiments designed for different aims. In Experiment 1, we aimed to assess whether or not there was a relationship between egg size and larval size-at-hatch. To do that, 82 sardine eggs were reared individually in 100-mL containers submerged in a water bath, maintaining the same temperature, salinity and light regime conditions as described below. Egg size was measured immediately after collection from the adult tank, and larvae size (total length) was measured at hatching.

To investigate the relationship between size-at-hatch and larval viability under optimal feeding conditions (Experiment 2), ~2000 sardine eggs were transferred to 30-L cylindrical tanks filled with seawater of salinity 35 and kept at 15 °C; these temperature and salinity conditions are the ones experienced during the peak spawning season for sardines off Portugal^57^. The light regime was kept at 16:8 light:dark, and gentle aeration was used to maintain steady water circulation and high oxygen concentrations. The sardine larvae were reared with two types of feeding conditions: a) optimal, which corresponded to those previously shown to translate into feeding and growth rates at saturated feeding levels^48^,^49^, and b) under starvation. The diet of sardine larvae in the rearing tanks with optimal feeding conditions comprised the dinoflagellate *Gymnodinium* sp., the rotifer *Brachionus* sp. and nauplii, copepodites and adults of the copepod *Acartia grani*. Details of the feeding regime are provided in^48^. The concentration of prey in the larval tanks was monitored daily by counting the prey left from the previous day and adding new prey to maintain the same concentration throughout the experimental period. Every day, the bottom of the tank was syphoned and dead larvae were counted to determine age-specific mortality rates.

In Experiment 3, the set-up was similar to the one in Experiment 2, but the larvae were kept only under starvation conditions, in order to assess the relationship between the size-at-hatch and the time until death of starved larvae.

### Egg and larvae size determinations, and otolith analysis

Sardine eggs from experiment 1 were measured individually after collection from the rearing tanks under a stereoscopic microscope (Zeiss STEMI 2000, magnification of 80×). Eggs were first inspected to be at an advanced stage of development, with the embryo with a well formed tail (Stage X^58^). Digital pictures of eggs were taken using the software Visilog Expert 6.300 and two types of measurements were done for each egg: egg total area and yolk sac area. At hatch, total length of all larvae was determined under the stereoscopic microscope.

In experiments 2 and 3, groups of larvae were randomly sampled from tanks to determine their size and conduct otolith analysis. Sardine larvae reared under optimal feeding conditions (Experiment 2) were sampled from 0 to 75 days post hatching (dph), whereas sardines reared under starvation (Experiment 3) were sampled until 10 dph (longest survival time was 11 dph). Sampling included 20 larvae at hatching, ≥10 individuals from 3 to 15 dph, and from 15 dph until the end of the experiment, 5–10 individuals were collected every 5 days. Each sampling day, larvae were immediately measured (total length) and preserved in formaldehyde (4%) for posterior otolith extraction.

Sardine otoliths were extracted using fine needles and both sagittae were removed and mounted on glass slides, using DPX. Otoliths were examined with an Olympus BX60 microscope. Examination and reading of the otoliths was carried out using 2000× for small otoliths and 1000× for large otoliths; resolution was 0.24 μm. A DP50 colour photo-camera was coupled to the microscope, and the Viewfinder Lite image-analyzer software was used to capture digital images of the otoliths. Otolith increments were measured with the ImageJ software. Increments were counted, beginning with the first clearly visible increment outside the core. Otoliths measurements included the total diameter and the diameter of the first check.

### Study of wild sardine larvae

**Figure 8:**
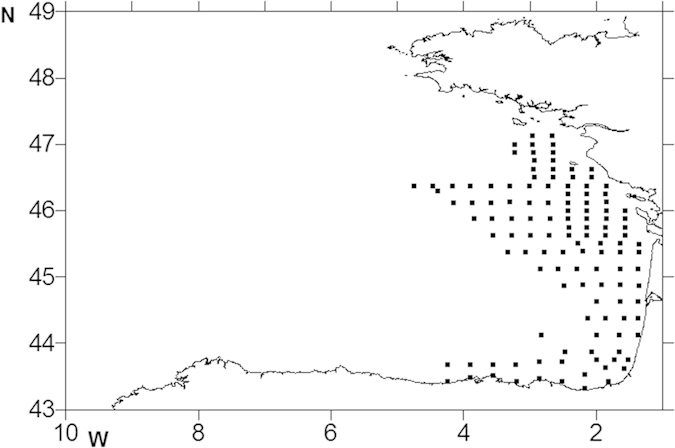
Sampling sites of European sardine (*Sardina pilchardus)* larvae in the Bay of Biscay. The map outline was generated using the software Surfer®8 (Golden Software, LLC).

To examine whether otolith size-at-hatch was related to larval survival in wild conditions, the otoliths of field-caught sardine larvae were analysed. Sardine larvae were collected on board the R/V Investigador from 6^th^ to 26^th^ May 2008 throughout 124 sampling stations distributed in the eastern region of the Bay of Biscay (Fig. 8). At each station, Bongo (336 μm) and MIK (1000 μm) nets were deployed and oblique tows from 5 m above the bottom to surface conducted to capture the fish larvae. Fish larvae samples were preserved in 80% Ethanol for posterior identification. Sardine larvae were identified in the samples, and then 504 well preserved and intact specimens were randomly selected and measured with 0.1 mm precision. Otoliths were extracted and analysed as described above except for the reading of the otoliths, which was done with 500× magnification and the size measurement of the first check (hatch check), for which the radius was determined instead of the diameter. Since the temperature and feeding history of field-caught larvae was not known, larval size-at-hatch was compared with larval total length instead of otolith-derived determinations of larval age.

### Statistical analyses

A linear regression model was applied to describe the relationship between laboratory-reared sardine larval length (total length) and otolith hatching check radius.

To infer the differential disappearance of larvae as a function of their initial size, a quantile regression analysis was carried out at respectively quantiles 0.10 and 0.90 of larvae’s hatching check diameter vs. their actual length. This analysis was carried on laboratory reared in optimal feeding conditions, under starvation and for field sardine larvae separately. 0.10 and 0.90 quantile regression coefficients are used to infer the differential mortality (disappearance from the batch) between small and large sized larvae at hatch, respectively. Quantile regression analysis was limited to the age interval where a sufficient number of larvae (minimum of 6 individuals) was sampled per length class, corresponding to all ages for starving larvae (0–10 dph) and from 0 to 20 dph for fed larvae in the laboratory, and from 6 to 22 mm for wild-caught larvae.

Daily mortality rates of sardine larvae hatched in the laboratory were calculated based on observed mortality of experiments conducted at 15 °C and a salinity of 35 psu. Cumulative mortalities were calculated by adding up daily rates from day 0 to day 55.

All statistical analyses and graphics were performed using Matlab 8.1 (R2013a) from Mathworks.

## Additional Information

**How to cite this article**: Garrido, S. *et al.* Born small, die young: Intrinsic, size-selective mortality in marine larval fish. *Sci. Rep.* **5**, 17065; doi: 10.1038/srep17065 (2015).

## Supplementary Material

Supplementary Information
