# Supplementary material for: Born small, die young: Intrinsic, size-selective mortality in marine larval fish
Source: Sci Rep. 2015 Nov 24;5:17065. doi: 10.1038/srep17065 (PMC4657020; doi:10.1038/srep17065)
Supplement: Supplementary Information [file srep17065-s1.pdf]

Supplementary Material

Born small, die young: Intrinsic, size-selective mortality in marine larval fish

Garrido, S.[1,2*], Ben-Hamadou, R.[3*], Santos A.M.[1], Ferreira, S.[1], Teodósio, M.A.[4], Cotano, U.[5], Irigoien, X.[5,#]., Peck, M.A.[6], Saiz, E.[7], and Ré, P.[2]

[1]Instituto Português do Mar e da Atmosfera, Av. Brasília s/n, 1449-006 Lisboa, Portugal.

[2]Marine and Environmental Sciences Centre, Faculdade de Ciências, Universidade de Lisboa, Campo Grande, 1749-016 Lisboa, Portugal.

[3]Department of Biological and Environmental Sciences, College of Arts and Sciences, Qatar University, PO Box 2713, Doha, Qatar.

[4]Centro de Ciências do Mar do Algarve, Universidade do Algarve. Campus de Gambelas, 8005-139 Faro, Portugal.

[5] Marine Research Unit – AZTI Foundation, Herrera Kaia, Portualdea z/g , 20110 Pasaia,, Spain.
[6] Institute for Hydrobiology and Fisheries Science, Hamburg University, Grosse Elbstrasse 133, 22765 Hamburg, Germany.

[7] Institut de Ciències del Mar - CSIC, Ps. Marítim de la Barceloneta 37-49, 08003 Barcelona, Spain.
# Present address: Red Sea Research Center, King Abdullah University for Science and Technology, 23955-6900 Thuwal, Saudi Arabia.

Table of contents

Table S1. Compiled information about regressions reported in Fig. 2, 4, 5, 6 and 7

Supplementary Table S1: Compiled information about regressions reported in Fig. 2, 4, 5, 6 and 7

Figures	Regression equations	Confidence interval (95% confidence bounds)		Goodness of fit	
		Regression coefficient (Slope)	Intercept	Adjusted R-square	One-tailed probability (*P*-value)
Figure 2A - Growth of sardine larvae reared in the laboratory under starvation	$Y = 0.254\ X + 3.86$	0.246 - 0.262	3.79 - 3.92	0.85	0.00007
Figure 4 – Relationship between otolith hatch check diameter (μm) and larvae size-at-hatch (total length, mm) for sardine larvae reared in the laboratory under optimal feeding conditions	$Y = 0.156\ X + 1.96$	0.114 - 0.198	1.52 - 2.4	0.25	0.0007
Figure 5 – Relation between sardine larvae age (days post-hatch) and otolith first increment check diameter (μm) for European sardine (*Sardina pilchardus*) larvae reared in the laboratory under optimal feeding conditions	10th percentile: $Y = 0.142\ X + 9.38$	0.039 - 0.244	9.28 - 9.48	0.6052	0.00003
	50th percentile: $Y = 0.088\ X + 11.29$	-0.014 - 0.190	11.19 - 11.39	0.5232	0.0084
	90th percentile: $Y = 0.035\ X + 13.2$	-0.103 - 0.173	13.09 - 13.30	0.393	0.013
Figure 6 – Relation between sardine larvae age (days post-hatching) and otolith first increment check diameter (µm) for European sardine (*Sardina pilchardus*) larvae reared in the laboratory under starvation.	10th percentile: $Y = 0.327\ X + 8.13$	0.224 - 0.429	8.03 - 8.23	0.694	0.000002
	50th percentile: $Y = 0.207\ X + 10.68$	0.105 - 0.309	10.58 - 10.78	0.723	$< 10^{-10}$
	90th percentile: $Y = 0.087\ X + 13.24$	-0.015 - 0.189	13.14 - 13.34	0.203	0.13
Figure 7 – Relation between sardine larvae age (Days post-hatching) and otolith first increment check diameter (µm) for larvae captured in the Bay of Biscay.	10th percentile: $Y = 0.12\ X + 3.8$	0.015 - 0.225	3.69 - 3.9	0.424	0.039
	50th percentile: $Y = 0.068\ X + 5.8$	-0.037 - 0.173	5.69 - 5.90	0.563	0.007
	90th percentile: $Y = 0.017\ X + 7.8$	-0.088 - 0.122	7.69 - 7.90	0.398	0.045
